# Computed Tomography-Based Radiomic Analysis for Preoperatively Predicting the Macrovesicular Steatosis Grade in Cadaveric Donor Liver Transplantation

**DOI:** 10.1155/2022/2491023

**Published:** 2022-01-22

**Authors:** Shengnan Ding, Weimin Yang, Xiaodong Sun, Yan Guo, Guangjie Zhao, Jinzhu Yang, Lei Zhang, Guoyue Lv

**Affiliations:** ^1^Department of Radiology, The First Hospital of Jilin University, Changchun, Jilin 130021, China; ^2^Department of Emergency, The First Hospital of Jilin University, Changchun, Jilin 130021, China; ^3^Department of Hepatobiliary and Pancreatic Surgery, The First Hospital of Jilin University, Changchun, Jilin 130021, China; ^4^GE Healthcare, 1 Niudun Road, Zhangjiang High Tech Zone, Shanghai 201203, China; ^5^Northeast University, Key Laboratory of Intelligent Computing in Medical Image, Ministry of Education, China

## Abstract

This study is aimed at determining the ability of computed tomography- (CT-) based radiomic analysis to distinguish between grade 0/1 and grade 2/3 macrovesicular steatosis (MaS) in cadaveric donor liver transplantation cases. Preoperative noncontrast-enhanced CT images of 150 patients with biopsy-confirmed MaS were analyzed retrospectively; these patients were classified into the low-grade MaS (*n* = 100, grade 0 or 1) and high-grade MaS (*n* = 50, grade 2 or 3) groups. Three-dimensional spherical regions of interest of 40 pixel (2.5 cm) in diameter were placed in the right anterior and left lateral segments of the liver. Thereafter, 300 regions of interest (ROIs) were segmented and randomly assigned to the training and testing groups at a ratio of 7 : 3. A total of 402 radiomic features were extracted from each ROI. For MaS classification, a radiomic model was established using multivariate logistic regression analysis. Clinical data, including age, sex, and liver function, were collected to establish the clinical model at the patient level. The performances of the radiomic and clinical models, i.e., the diagnostic discrimination, calibration, and clinical utilities, were evaluated. The radiomic model, with seven selected features, depicted a good discrimination with an area under the receiver operating characteristic curve (AUC) of 0.907 (95% confidence interval (CI): 0.869–0.940) in the training cohort and 0.906 (95% CI: 0.843–0.959) in the testing cohort. The calibration curve revealed good agreement between the predicted and observed probabilities in the training and testing cohorts (both *P* > 0.05 in the H-L test). Decision curve analysis revealed that the radiomic model was more beneficial than the treat-all or treat-none schemes for predicting the MaS grade. Alanine transaminase and gamma-glutamyl transferase were used for building the clinical model, and the AUC was 0.784 in the total cohort. The CT-based radiomic model outperforming the conventional clinical model could provide an important reference for MaS grading in cadaveric liver donors.

## 1. Introduction

The insufficient number of grafts available for liver transplantation (LT) has increased mortality among patients on waiting lists and triggered the use of organs from marginal donors. One of the most common marginal donors for LT are those with hepatic steatosis. Hepatic steatosis is considered one of the most common disorders; this is mostly due to the growing incidence of nonalcoholic fatty liver disease [1–3]. The presence of significant steatosis is correlated with the progression of initial poor graft function or primary graft nonfunction [4]. Available evidence reveals an increased risk of poor graft outcomes in patients with moderate-to-severe steatotic livers [2, 5]. From a pathological perspective, steatosis can be categorized into microvesicular, macrovesicular, and mixed forms. We have focused on macrovesicular steatosis (MaS) in this study because it is a major cause of graft failure in donor livers [6]. The use of grafts with mild steatosis (MaS content < 30%) is safe; however, grafts with moderate-to-severe steatosis (MaS content > 30%) are not recommended for use. Therefore, accurate grading of MaS is important in the evaluation of donors before LT.

Although liver biopsy is the gold standard for the assessment of MaS, it is limited by sampling variability, costs, invasiveness, and severe complications (such as mortality, bleeding, and pain) [7]. Among noninvasive imaging methods, emergency computed tomography (CT) is most commonly performed before LT because cadaveric liver donors are mostly critically ill patients with a history of ischemic or hemorrhagic stroke and trauma; a thorough assessment in these donors is not possible. Therefore, time constraints and possible hemodynamic instability make it impossible to use techniques, such as magnetic resonance imaging (MRI). However, accurate assessment of the MaS severity using traditional CT, such as liver densities in Hounsfield units (HU) and the CT liver-to-spleen ratio, is difficult because liver abnormalities, such as edema, inflammation, iron overload, ischemia, and ingestion of certain drugs (e.g., amiodarone) [8–10], are more common in cadaveric liver donors.

Radiomic analysis can be used to extract a large amount of feature information from acquired images by mining and analyzing the feature data to establish an accurate diagnosis of the disease. Previous studies have suggested that CT or MRI texture analysis can effectively predict nonalcoholic steatohepatitis [11] and assess hepatic fibrosis [12–15]. In addition, other studies have detected MaS in living liver donors by noninvasive imaging modalities, such as MRI and the controlled attenuation parameter [10, 16–18]. However, few studies have focused on the efficiency of radiomic features on noncontrast-enhanced CT (NECT) images for MaS grading in cadaveric liver donors. Thus, this study is aimed at determining the ability of CT-based radiomic analysis to predict grade 0/1 and 2/3 MaS in cadaveric liver donors having undergone liver biopsy as the gold standard.

## 2. Materials and Methods

### 2.1. Ethical Considerations

This study was approved by the Ethics Committee of The First Hospital of Jilin University (2020-324). The requirement of informed consent was waived due to the retrospective nature of the study.

### 2.2. Patients

**Figure 1:**
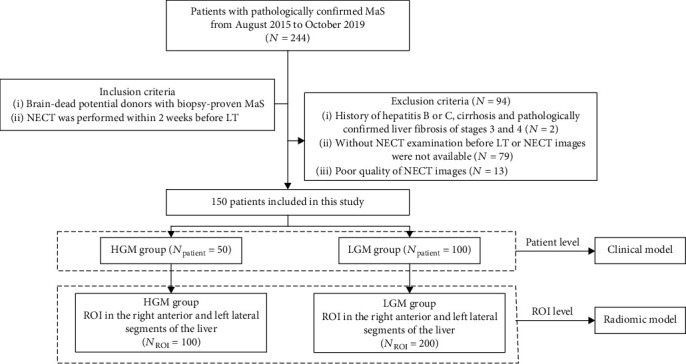
Flow diagram of the patient enrollment. MaS: macrovesicular steatosis; NECT: noncontrast-enhanced computed tomography; HGM: high-grade MaS; LGM: low-grade MaS; ROI: region of interest.

Brain-dead potential donors with biopsy-proven MaS encountered from August 2015 to October 2019 were retrospectively included in this study. As fibrosis influences the CT texture features [12–15], patients were excluded if they had a history of hepatitis B or C, cirrhosis, or stage 3 or 4 liver fibrosis. The inclusion and exclusion criteria are presented in Figure 1. Based on these criteria, 150 patients were ultimately included in this study. Donors with grade 2 and 3 MaS were grouped into the “high-grade MaS” (HGM) group (*n* = 50), whereas those with grade 0 and 1 MaS were grouped into the “low-grade MaS” (LGM) group (*n* = 100).

### 2.3. Liver Biopsy and Pathology

Biopsy specimens were obtained by wedge resection. The histological grading of MaS was performed by two experienced pathologists blinded to the clinical data and study design. The extent of MaS was evaluated and reported semiquantitatively as a precise percentage of the hepatocytes involved; it was graded as S0: absent (0%–5%), S1: mild (>5%–30%), S2: moderate (>30%–60%), or S3: severe (>60%) [19].

### 2.4. CT Image Acquisition

CT examinations were performed using either a 256-detector row scanner (Philips Brilliancei, 14 patients in Israel; GE Medical Systems, four patients in the USA) or a 64-detector row scanner (Philips Brilliancei, 128 patients in Israel; GE Medical Systems, four patients in the USA). All scanners used identical settings: 120 kV, 150–250 mA (depending on the body weight), 0.5 s per rotation, pitch: 0.8–1.0 : 1, matrix: 512, and reconstruction thickness: 1 mm.

### 2.5. Radiomic Model Building

**Figure 2:**
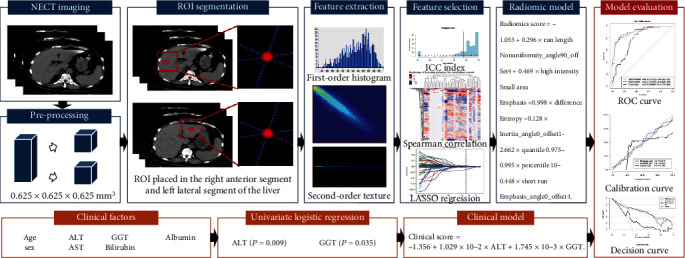
The flowchart of this study. (1) Radiomic model (blue): after preprocessing, a three-dimensional segmentation of an ROI with a 40-pixel (2.5 cm) diameter on CT images is performed. Features are extracted from the ROI, including the first-order histogram and second-order texture features. The ICC index, Spearman correlation, and LASSO are used for the radiomic feature selection. The radiomics signature is built based on the remaining features using logistic regression analysis. (2) Clinical model (orange): after univariate logistic regression analysis, ALT and GGT (variables with *P* < 0.05) are used for building the clinical model, weighted by their respective coefficients. (3) Model evaluation (red): the performances of the radiomic model and clinical model are evaluated with ROC, calibration, and decision curves. NECT: noncontrast-enhanced computed tomography; ICC: intraclass correlation coefficient; LASSO: least absolute shrinkage and selection operator; ALT: alanine aminotransferase; AST: aspartate aminotransferase; GGT: gamma-glutamyl transferase; ROC: receiver operating characteristic; ROI: region of interest.

The flowchart in Figure 2 presents the development of the radiomic model and clinical model used for MaS grade prediction and model evaluation. The image biomarker standardization initiative was regarded as the reference and taken into consideration in most of the image preprocessing, feature extraction, and selection procedures [20]. The steps involved in the radiomic and clinical model building are described in the following paragraphs.

#### 2.5.1. Preprocessing and ROI Segmentation

Images were first preprocessed using the Artificial Intelligence Kit Software (A.K. Software; version 3.2.5, GE Healthcare, China) and then exported to the open-source ITK-SNAP software (version 3.8.0; http://www.itksnap.org) for segmentation. For preprocessing, a linear interpolation algorithm (which could construct new data points according to the range of a discrete set of known data points) was used to resample the voxels from 0.625 × 0.625 × 1.000 mm^3^ to isotropous with an *x*-spacing, *y*-spacing, and *z*-spacing of 0.625 mm, 0.625 mm, and 0.625 mm, respectively, i.e., 0.625 × 0.625 × 0.625 mm^3^. Data preprocessing was performed to ensure that the voxels were isotropic; this would prevent variability in the histogram and texture features among different voxel sizes. Subsequently, a three-dimensional spherical region of interest (ROI), having a 40-pixel (2.5 cm) diameter, was placed in the right anterior and left lateral segments of the liver at the level of the porta hepatis (as depicted in Figure 2). All segmentations were conducted by a radiologist (DSN) with a 5-year experience in abdominal CT. To assess the reproducibility of the features obtained from the ROIs segmented by different readers, 30 patients (10 from the HGM group) were randomly selected for resegmentation by another abdominal radiologist with a 10-year experience. Both radiologists were blinded to the pathological findings.

From the 150 enrolled patients, 300 ROIs (HGM group: 100 ROIs, LGM group: 200 ROIs) were included in this study. In total, 210 ROIs (70%) were randomly included in the training cohort for the development of the model and the remaining 90 (30%) were assigned to the testing cohort to verify the performance of the radiomic model.

#### 2.5.2. Feature Extraction

Feature extraction was performed using the A.K. Software. From each ROI in the NECT images, 402 radiomic features were extracted. These included the following: (1) first-order histogram features describing the HU distribution of the ROIs (*n* = 42), (2) gray-level cooccurrence matrix- (GLCM-) based features describing the probability of given voxel pairs occurring next to each other (*n* = 144), (3) gray-level size zone matrix- (GLSZM-) based features describing the size and number of the connected regions of all gray scales in the image (*n* = 11), (4) gray-level run length matrix- (GLRLM-) based features enumerating the probability of identical voxel values being continuously next to each other (*n* = 180), (5) Haralick texture features (*n* = 10), and (6) formfactor features (*n* = 15). Details of the extracted radiomic features are presented in Supplementary Figure 1.

#### 2.5.3. Feature Selection

To ensure the repeatability of the model, we calculated the intraclass correlation coefficient (ICC) of the radiomic features extracted by the two readers from the randomly selected 30 patients. Features with ICC > 0.75 were considered to have a good reproducibility [21] and were retained for subsequently analysis. All features were then standardized and normalized in the training dataset, and the same procedure was applied in the testing dataset. All feature selection techniques were applied in the training dataset as follows. First, a Spearman correlation analysis was performed to eliminate redundant features that were highly correlated with other features (∣*r* | >0.9). Then, a least absolute shrinkage and selection operator (LASSO) regression analysis was performed to select the features; the coefficients of useless features were shrunk to zero with the regulation parameter *λ*, using 10-fold crossvalidation.

#### 2.5.4. Clinical Model Building

Data on the clinical factors, including age, sex, and liver function, were collected at the patient level. Variables with *P* < 0.05 in the univariate analysis were included in the multivariate logistic regression analysis to identify the independent clinical risk factors associated with MaS grading. The clinical model was established based on the chosen independent risk factors by applying multivariate logistic regression.

#### 2.5.5. Radiomic Model Building

A multivariate logistic regression method was used to establish a classification model for MaS grading based on the selected features. Then, a radiomic signature was produced with the selected features using a linear function and the signature served as an independent variable in the sigmoid function.

### 2.6. Performance Evaluation

The performance of the radiomic model was evaluated with discrimination, calibration, and clinical application in both the training and testing cohorts. The performance of the clinical model was evaluated using the same parameters in the total cohort.

#### 2.6.1. Discrimination

The receiver operating characteristic (ROC) curve was plotted to evaluate the diagnostic performances of the radiomic model and clinical model, and the area under the ROC curve (AUC) was calculated with its 95% confidence interval (CI). The optimal cutoff value of the radiomic model was obtained based on the maximum Youden index in the training cohort; it was then applied in the testing cohort. The specificity, sensitivity, positive predictive value, negative predictive value, and diagnostic accuracy were calculated based on the optimal cutoff values in both the training and testing cohorts. The Delong test was used to compare the differences in the AUCs between the training and testing datasets and between the clinical and radiomic models.

#### 2.6.2. Calibration

Calibration curves were plotted to evaluate the agreement between the observed outcome frequencies and predicted probabilities of the model. The Hosmer–Lemeshow test was performed to determine the goodness of fit; a *P* value > 0.05 was considered to indicate a well-calibrated fit.

#### 2.6.3. Clinical Usefulness

A decision curve analysis (DCA) was performed to assess the clinical usefulness of the model by quantifying the net benefits at different threshold probabilities.

### 2.7. Statistical Analyses

All statistical analyses were conducted with the R Studio software (version 1.2.1335). A two-sided *P* value < 0.05 was considered statistically significant. Categorical variables (sex) were compared using the *χ*^2^ test, and continuous variables were compared using the *t*-test for variables with a normal distribution or the Mann–Whitney *U* test for variables with an abnormal distribution. The ICC was calculated using the “lme4” package. The “glmnet” package was used for LASSO regression. The “rms” package was used for multivariate logistic regression. The “pROC” package was used to plot the ROC curves and to measure the AUCs. The “ModelGood” package was used for plotting the calibration curves and for the Hosmer–Lemeshow test. The “dca.R” package was used to perform the DCA.

## 3. Results

### 3.1. Patient Characteristics

The HGM group (*n* = 50) comprised 19 patients with grade 2 MaS and 31 with grade 3 MaS. The LGM group (*n* = 100) comprised four patients with grade 0 MaS and 96 with grade 1 MaS. Mortality in 53.3%, 39.3%, and 7.4% of all cases resulted from ischemic or hemorrhagic stroke, trauma, and other causes, respectively. Comparisons of clinical characteristics are presented in Table 1. The aspartate aminotransferase (AST), alanine aminotransferase (ALT), and gamma-glutamyl transferase (GGT) levels were significantly higher in the HGM group than in the LGM group, with *P* values of 0.005, <0.001, and <0.001, respectively.

### 3.2. Clinical Model

The AST, ALT, and GGT levels were significantly different between the LGM and HGM groups (Table 1). Thus, they were considered for the univariate and multivariate regression analyses. Univariate analysis revealed that ALT and GGT levels were significant risk factors (*P* < 0.05 for both); therefore, these were included in the multivariate logistic regression analysis. Multivariate analysis further indicated that the ALT level (odds ratio (OR) = 1.010; 95% CI: 1.002–1.019; *P* < 0.05) was an independent clinical risk factor for the prediction of the MaS grade, as shown in Table 2. The ALT and GGT levels were used for building the clinical model. Thereafter, the clinical score was calculated via the following formula:

(1) $$\begin{array}{c} \text{Clinical Score}=-1.356+1.029\times 10^{-2}\times \text{ALT}+1.745\times 10^{-3}\times \text{GGT} \end{array}$$

**Figure 3:**
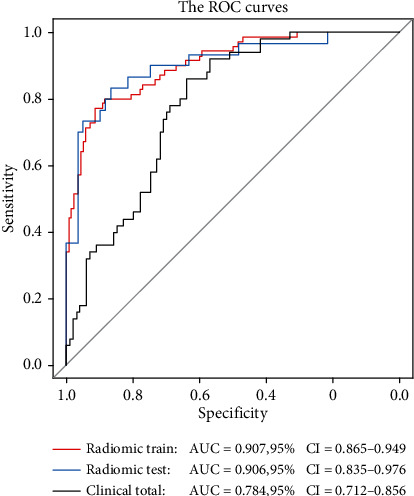
ROC curves of the radiomic model in the training (red) and testing (blue) cohorts and of the clinical model in the total cohort (black). The radiomic model had a significantly better performance than the clinical model, with *P* < 0.05 (DeLong test; radiomic model in the training set vs. clinical model in the total set, *P* = 0.004; radiomic model in the testing set vs. clinical model in the total set, *P* = 0.019). Meanwhile, the performance of the radiomic model did not differ significantly between the training and test cohorts (DeLong test; *P* = 0.978). ROC: receiver operating characteristic; AUC: area under the curve.

**Figure 4:**
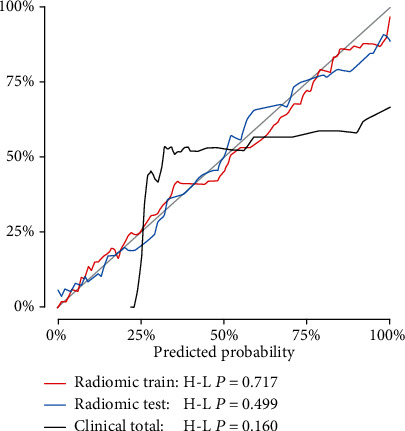
Calibration curves of the radiomic model in the training (red) and testing (blue) cohorts and of the clinical model in the total cohort (black). The gray solid lines represent perfect prediction; the closer the lines to the gray reference line, the better the model fit.

**Figure 5:**
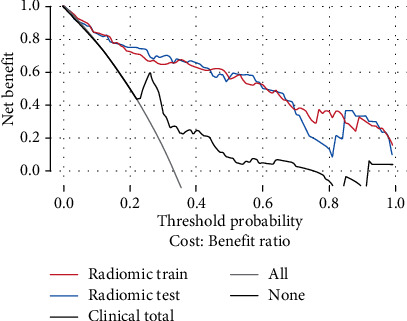
Decision curves of the radiomic model in the training (red) and testing (blue) cohorts and of the clinical model in the total cohort (black). The *y*-axis represents the net benefit of the patients. The net benefit is determined by calculating the difference between the expected benefit and expected harm associated with each proposed model (net benefit = true-positive rate – [false-positive rate × weighting factor], where the weighting factor = threshold probability/[1 − threshold probability]). The gray line represents the assumption that all livers had HGM (the treat-all scheme). The black horizontal line represents the assumption that all livers had LGM (the treat-none scheme). Using the radiomic model to predict the MaS grade added more benefits as compared with using the clinical model at almost any given threshold probability.

The diagnostic performance of the clinical model is presented in Table 3, and the corresponding ROC curve is illustrated in Figure 3. The Delong test revealed significant differences in the performances of the clinical model and radiomic model (*P* value for both < 0.05). The calibration and decision curves are illustrated in Figures 4 and 5, respectively.

### 3.3. Radiomic Model

Four hundred and two features were extracted from the NECT images; after ICC analysis, Spearman correlation analysis, and LASSO regression, seven features were selected. These included two histogram-based, two GLCM-based, two run length matrix- (RLM-) based, and one GLSZM-based features. Detailed information on feature selection is available in (available here) Supplementary Material A. Multivariate logistic regression analysis was performed to build a radiomic signature based on the selected seven features. The radiomics score was calculated using the following formula:

(2) $$\begin{array}{c} \text{Radiomics score}=-1.053+0.296\times \text{RunLengthNonuniformity}\_\text{angle}90\_\text{offset }4+0.469\times \text{HighIntensitySmallAreaEmphasis}+0.998\times \text{differenceEntropy}-0.128\times \text{Inertia}\_\text{angle}0\_\text{offset}1-2.662\times \text{Quantile}0.975-0.995\times \text{Percentile}10-0.448\times \text{ShortRunEmphasis}\_\text{angle}0\_\text{offset}4 \end{array}$$

The diagnostic performance of the radiomic signature in the training and testing cohorts is depicted in Table 3. The ROC curves for the training (a) and testing (b) datasets are illustrated in Figure 3. There were no significant differences between the AUCs of the radiomic models in the training and test cohorts (DeLong test; *P* value = 0.978).

The calibration curve for the radiomic model is demonstrated in Figure 4, which reveals a good agreement between the predicted probability and observed probability in both the training and testing cohorts. Moreover, the *P* value was > 0.05 according to the Hosmer–Lemeshow test.

The decision curve for the radiomic model is illustrated in Figure 5. Using the radiomic model to predict the MaS grade added greater benefits as compared with using the treat-all scheme or treat-none scheme at any given threshold probability in the training cohort. For threshold probabilities > 10%, using the radiomic model to predict the MaS grade added greater benefits as compared with using the treat-all scheme or treat-none scheme in the testing cohort.

## 4. Discussion

In this study, we developed a radiomic signature based on NECT and the capability of this radiomic signature for estimating the MaS grade was found to be satisfactory. The radiomic model was superior to the clinical model. This noninvasive method of assessing the MaS grade only relied on NECT; therefore, this method could provide an important reference for donors before LT.

Currently, organ shortage is a prominent problem in clinical settings. In China, donation after cardiac death has become the main method for organ donation because of its special social environment. The prevalence of obesity in the population has significantly increased the incidence of liver steatosis. Available evidence has revealed that more than 30% of the cases with hepatic steatosis are associated with poor post transplantation graft outcomes [5]. Although MaS grading is important in donors, liver biopsy or other noninvasive imaging methods are not always safe, satisfactory, or practical in these individuals. In our study, we developed and validated a radiomics approach based on NECT for the prediction of the MaS grade in cadaveric liver donors; the approach demonstrated good discrimination, calibration, and clinical utilities.

Previous studies have reported that MRI can predict the MaS grade [22, 23]. In recent years, the MRI proton density fat fraction has been used as a noninvasive and quantitative measure for accurately classifying the hepatic steatosis grade [22, 23]. However, compared with CT, MRI is more time-consuming and expensive. Furthermore, critically ill patients often need a ventilator, which limits the use of MRI. Abdominal ultrasonography is the most widely available modality for detecting steatosis, and its main disadvantage is its operator dependency. It has an acceptable level of sensitivity, although it is not suitable for providing objective quantitative data on steatosis in donors [4, 9]. In this study, we used NECT instead of contrast-enhanced CT or other noninvasive imaging methods because deceased liver donors are mostly critically ill patients who are usually examined by NECT before LT (which is essential for the diagnosis of MaS). Moreover, the radiomic features in contrast-enhanced CT images are usually influenced by the injection rate, patient's circulation, and the phase of enhancement.

In this study, we also analyzed the clinical features. After univariate and multivariate regression analyses, the ALT and GGT levels were used for building the clinical model. The AUC of the clinical model was less than that of the radiomic signature model (0.784 vs. 0.907, respectively). Therefore, the radiomic model could better predict the MaS grade as compared with the clinical model. This is because the clinical model cannot be used for quantitative research and data on some clinical factors associated with hepatic steatosis (such as the blood lipid levels and body mass index) are usually not available for deceased liver donors.

The radiomic signature for MaS grading comprised seven imaging features that were extracted from the three-dimensional NECT images. Percentile10 and quantile0.975 were the histogram parameters. Inertia_angle0_offset1 and difference entropy were the GLCM parameters. HighIntensitySmallAreaEmphasis was one of the GLSZM-based feature parameters. RunLengthNonuniformity_angle90_offset4 and ShortRunEmphasis_angle0_offset4 were the RLM parameters. These features describe the distribution of the voxel intensities within an image and nonuniformity of the grayscale and length, which could potentially reflect the MaS heterogeneity. For example, the larger the value of the difference entropy, the rougher the texture and higher the heterogeneity of MaS. Therefore, the observed abnormalities in the liver images may be clinically associated with the MaS grade. Because fatty liver comprises diffused lesions, unlike a tumor, sketching its edges is difficult; therefore, we chose a spherical ROI of a fixed size to reduce the differences caused by human factors. As a result, no morphological features that differed statistically between the levels were retained.

Nevertheless, this study has some limitations. First, it is a retrospective study; thus, data on some clinical factors (such as the blood lipid levels, blood glucose levels, and body mass index) were not available. This is because cadaveric liver donors are mostly critically ill patients in whom thorough examinations are not performed before LT due to insufficient time. Further prospective research is needed to assess nomograms based on the selected radiomic signature and clinical characteristics for the prediction of the MaS grading performance. Furthermore, given the retrospective study design, our ROI profile and pathological findings may not match exactly. Therefore, ROI segmentation corresponding to the pathological sampling with further prospective validation is required to assess the predictive ability of the radiomic model in the future. Second, because fatty liver comprises diffuse lesions and sketching its edge may be difficult, a spherical ROI of fixed size was chosen to improve repeatability. However, the vessels and liver parenchyma have similar densities in a fatty liver. Therefore, it is difficult to identify vessels in ROIs that may have contained some portions of a vessel. At the same time, the morphological features may have been overlooked. In future studies, we will use other ROI segmentation methods for further verification. The third limitation is the small and imbalanced sample size. We chose the right anterior and left lateral segments because these are typically selected for biopsy in cadaveric liver donors. Given that the right anterior and left lateral segments of the liver had similar histologic MaS grades in our study, 300 ROIs from the 150 patients were included to expand the sample size. Our sample ratio of 1 : 2 does not exceed the limit of 1 : 3 prescribed as the upper limit of the distribution ratio of the sample size by multifactor logistic regression modeling; the results of the linear model may be affected if the limit is exceeded [24]. The model effectiveness can be improved by using the synthetic minority oversampling technique (SMOTE). However, considering the great retention of the original data, the SMOTE was not adopted to amplify the data. Finally, the radiomics quality score of our radiomic procedure was 16 (maximum value = 36), corresponding to a percentage of 44.4% [25]. The quality of our study may still have limitations that affect the reproducibility of the results. A more standardized methodology is required in the radiomics workflow, especially in terms of the study design, cost-effective analysis, and open science data, to translate the results to clinical applications.

## 5. Conclusions

Radiomic analysis of NECT images could provide an important reference for predicting grade 0/1 and 2/3 MaS in patients before LT. It is noninvasive and can achieve a satisfactory preoperative prediction of the individual MaS grade.

## Figures and Tables

**Table 1:** Clinical characteristics of the patients with MaS.

Characteristics	LGM (*n* = 100)	HGM (*n* = 50)	*P* value
Age (years)	46.94 ± 10.98	48.28 ± 10.50	0.501
Sex			0.882
Male	81 (81.00)	41 (82.00)	
Female	19 (19.00)	9 (18.00)	
ALT (IU/L)	21.25 (15.73, 34.03)	36.40 (26.28, 65.68)	<0.001^∗^
AST (IU/L)	41.15 (30.18, 56.45)	57.05 (36.38, 94.80)	0.005^∗^
GGT (IU/L)	27.00 (16.50, 60.55)	54.05 (32.15, 166.53)	<0.001^∗^
Bilirubin (*μ*mol/L)	17.86 ± 10.50	18.94 ± 11.21	0.564
Albumin (g/L)	36.72 ± 8.39	36.50 ± 10.53	0.888

Continuous variables are expressed as means ± standard deviations or as medians (25%, 75%), as appropriate. Categorical variables are presented as numbers (%). The ALT, AST, and GGT levels are significantly different between the two groups. ALT: alanine aminotransferase; AST: aspartate aminotransferase; HGM: high-grade MaS; GGT: gamma-glutamyl transferase; LGM: low-grade MaS; MaS: macrovesicular steatosis. ∗ indicates significance with *P* < 0.05.

**Table 2:** Univariate and multivariate logistic regression analyses of the clinical factors.

	Univariate logistic regression		Multivariate logistic regression	
Characteristics	OR (95% CI)	*P* value	OR (95% CI)	*P* value
ALT (IU/L)	1.012 (1.003–1.021)	0.009^∗^	1.010 (1.002–1.019)	0.021∗
AST (IU/L)	1.005 (1.000–1.010)	0.066	—	—
GGT (IU/L)	1.002 (1.000–1.004)	0.035^∗^	1.002 (1.000–1.004)	0.070

Variables with *P* < 0.05 in the univariate analysis were included in the multivariate logistic regression analysis. ALT: alanine aminotransferase; AST: aspartate aminotransferase; GGT: gamma-glutamyl transferase; OR: odds ratio; CI: confidence interval. ∗ indicates significance with *P* < 0.05.

**Table 3:** Diagnostic performances of the radiomic model and clinical model.

Performance	Radiomic model (ROI level)		Clinical model (patient level)
	Training cohort (*n* = 210)	Testing cohort (*n* = 90)	Total cohort (*n* = 150)
AUC (95% CI)	0.907 (0.869–0.940)	0.906 (0.843–0.959)	0.784 (0.719–0.839)
Sensitivity (%)	75.7	76.7	84.0
Specificity (%)	91.4	88.3	64.0
Accuracy (%)	86.2	84.4	70.7
Precision (%)	81.5	76.7	53.8
Positive predictive value (%)	81.5	76.7	53.8
Negative predictive value (%)	88.3	88.3	88.9

AUC: area under the curve; ROI: region of interest.

## Data Availability

The data used to support the findings of this study are available from the corresponding author upon request.

## Supplementary Materials

Supplementary materials Supplementary Figure 1: Details of the 402 radiomic features. Supplementary Material A: Detailed information regarding feature selection. LASSO,: least absolute shrinkage and selection operator. Figure A1.: Graph shows mean square error on each fold for the LASSO model. LASSO,: least absolute shrinkage and selection operator. Figure A2.: Graph shows the LASSO path plot of the model in the training samples. LASSO,: least absolute shrinkage and selection operator.
