# Supplementary material for: Computed Tomography-Based Radiomic Analysis for Preoperatively Predicting the Macrovesicular Steatosis Grade in Cadaveric Donor Liver Transplantation
Source: Biomed Res Int. 2022 Jan 22;2022:2491023. doi: 10.1155/2022/2491023 (PMC8800621; doi:10.1155/2022/2491023)
Supplement: Supplementary materials — Supplementary Figure 1: Details of the 402 radiomic features. Supplementary Material A: Detailed information regarding feature selection. LASSO,: least absolute shrinkage and selection operator. Figure A1.: Graph shows mean square error on each fold for the LASSO model. LASSO,: least absolute shrinkage and selection operator. Figure A2.: Graph shows the LASSO path plot of the model in the training samples. LASSO,: least absolute shrinkage and selection operator. [file 2491023.f1.docx]

**Supplementary Materials**

**Supplementary Figure 1: Details of the 402 radiomics features**

**
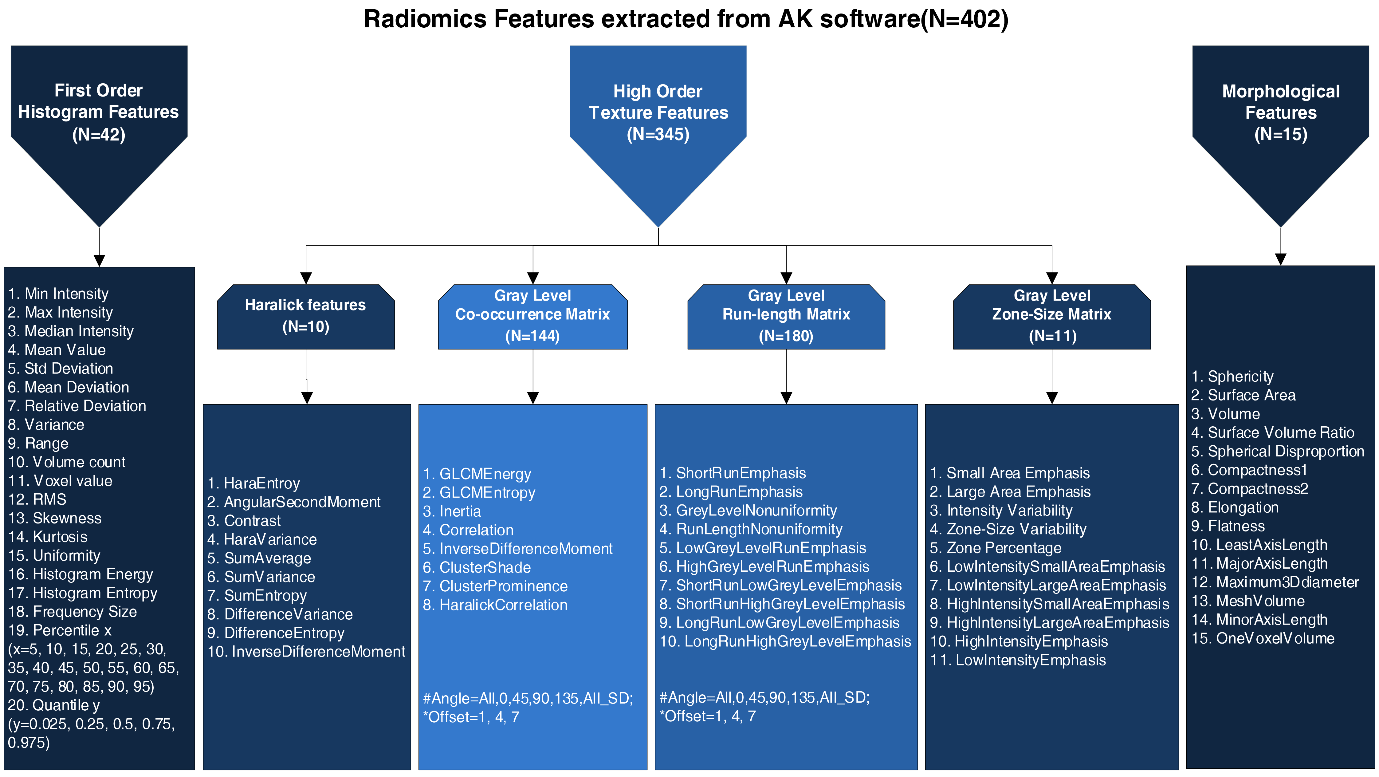
**

**GLCMEnergy, gray-level co-occurrence matrix energy; GLCMEntropy, gray-level co-occurrence matrix entropy.**

**Supplementary Material A: Detailed information regarding feature selection. LASSO, least absolute shrinkage and selection operator.**

**Detailed information on feature selection**

Step 1: The selective method was Spearman correlation analysis. The threshold value was 0.9, and the remaining feature number was 46.

Step 2: The selective method was LASSO. The mean square error on each fold for the LASSO model is illustrated in Appendix **Figure A1**. The LASSO path plot of the model in the training samples is shown in Appendix **Figure A2**. The remaining feature number was 7. The remaining features were as follows:

['RunLengthNonuniformity_angle90_offset4'];

['HighIntensitySmallAreaEmphasis'];

['differenceEntropy'];

['Inertia_angle0_offset1'];

['Quantile0.975'];

['Percentile10'];

['ShortRunEmphasis_angle0_offset4'].





**Figure A1.** Graph shows mean square error on each fold for the LASSO model. LASSO, least absolute shrinkage and selection operator.


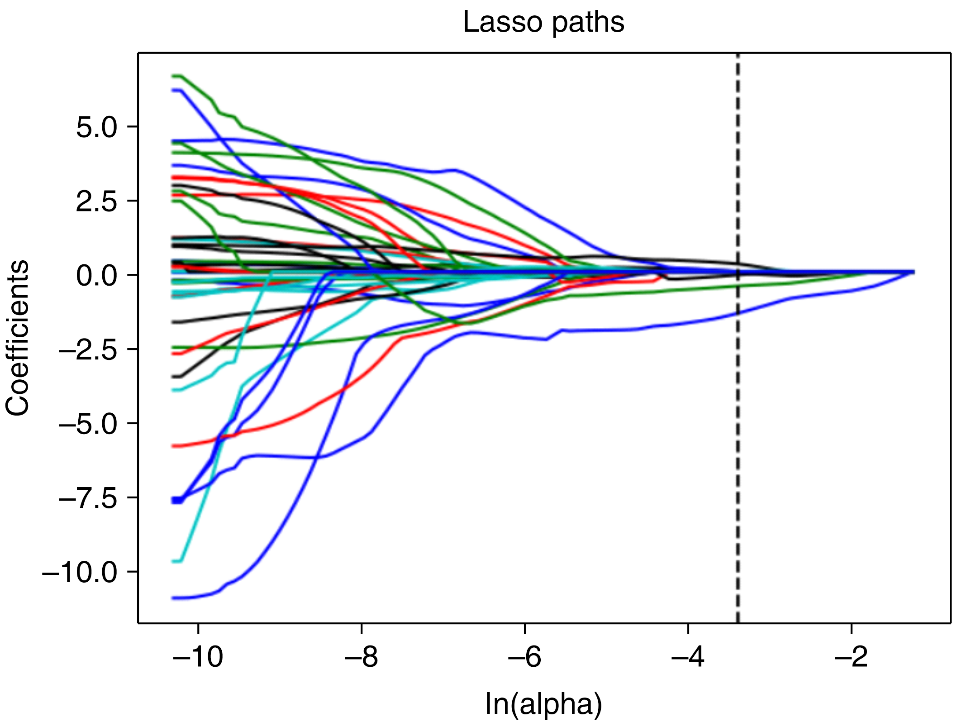


**Figure A2.** Graph shows the LASSO path plot of the model in the training samples. LASSO, least absolute shrinkage and selection operator.
